# Bioinformatics analysis and identification of hub genes associated with female acute myocardial infarction patients by using weighted gene co-expression networks

**DOI:** 10.1097/MD.0000000000033634

**Published:** 2023-04-28

**Authors:** Kun Jiao, Ping Su, Yubao Feng, Changqing Li

**Affiliations:** a Ordos Central Hospital Cardiology Department, Ordos, China.

**Keywords:** acute myocardial infarction, differentially expressed genes, female, IL-17 signaling pathway, inflammatory factors

## Abstract

To explore potential biomarkers of acute myocardial infarction (AMI) in females by using bioinformatics analysis. In this study, we explored potential biomarkers of AMI in females using bioinformatics analysis. We screened a total of 186 differentially expressed genes from the Gene Expression Omnibus. In the study, we found that weighted gene co-expression network analysis explored the co-expression network of genes and identified key modules. Simultaneously, we chose brown modules as key modules related to AMI. In this study, Gene Ontology and Kyoto Encyclopedia of Genes and Genomes pathway enrichment analysis revealed that genes in the brown module were mainly enriched in “heparin” and ‘complementation and coagulation cascade. Based on the protein-protein interaction network, we identified S100A9, mitogen-activated protein kinase (MAPK) 3, MAPK1, MMP3, interleukin (IL)-17A, and HSP90AB1 as hub gene sets. Whereas, polymerase chain reaction results showed that S100A9, MAPK3, MAPK1, MMP3, IL-17A, and HSP90AB1 were highly expressed compared with the control group. The IL-17 signaling pathway associated with an inflammatory response may be a potential biomarker and target for the treatment of women with myocardial infarction.

## 1. Introduction

The Heart Disease and Stroke Statistical Study (2020 edition) have revealed that cardiovascular disease (CVD) remains the leading cause of death globally, accounting for 17.8 million (95% confidence interval, 17.5–18.0 million) deaths worldwide in 2017, which might be a significant increase the global health burden.^[1]^ Acute myocardial infarction (AMI) is one of the most serious CVDs, which is characterized by a sudden reduction in blood flow to the myocardium and high morbidity and mortality.^[2]^ The current epidemiological studies have indicated that females with AMI may have a significantly higher risk of death and morbidity after myocardial infarction (MI) than men of the same age.^[3,4]^ But so far, the reasons for the increased risk in female patients are unclear. Recent studies have shown that inflammatory markers, such as interleukin (IL), might be elevated 1 month after MI in young females than in males.^[5]^ Relevant studies have shown that poor prognosis in women may be associated with an increased risk of recurrent cardiovascular events.^[6]^

Previous studies have shown that inflammation triggered by the immune system plays an indispensable role in the pathophysiological process of coronary heart disease.^[7]^ Related studies have pointed out that overexpression of the inflammatory mediator miR146a can decline cardiac dysfunction by reducing the level of inflammatory markers.^[8]^ Various evidence has also indicated that inflammation-related cytokines and chemokines are involved in the occurrence and progression of MI, such as IL-4, IL-6, IL-9, IL-13, IL-35, TNF-a, IFN- g, etc.^[9–12]^

Furthermore, a clinical study by Alsahly et al^[13]^ showed that the incidence rate of AMI in women was lower than that in men before menopause, but after menopause, the incidence rate of the former showed an obvious upward trend, which was due to the influence of female estrogen levels and the blood purification caused by regular intrauterine blood excretion, thus reducing the incidence rate of MI. Additionally, it also revealed that whether the high mortality and incidence rate of female MI after menopause is related to inflammatory reaction is still unclear. Therefore, it is urgent to find key genes or pathways to identify and explore specific mechanisms through experimental research. In this study, we screened the IL-16 signal pathway, which is closely related to inflammatory response, which does not provide a good basis for further research on the above issues.

In the present study, we reanalyzed the GSE95368 gene expression profiles of 12 female AMI patients and 6 controls. Among them, the differentially expressed genes (DEGs) were obtained by the “limma” package^[14]^ in R software. We performed Gene Ontology (GO) and Kyoto Encyclopedia of Genes and Genomes (KEGG) pathway enrichment analyses in key multiple organ dysfunction syndrome. Simultaneously, we determined and verified the pivot genes in the key modules by quantitative real-time polymerase chain reaction (RT-PCR). This study provided a research basis for exploring the potential regulatory targets and possible regulatory mechanisms for females with AMI and provided new ideas for the treatment of the disease.

## 2. Materials and methods

### 2.1. Material collection

**Figure 1.**
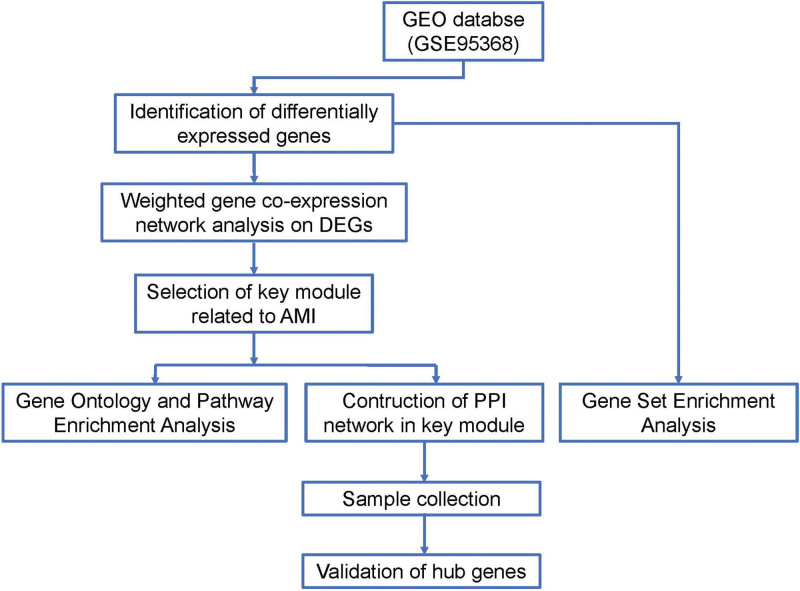
Schematic diagram of the process.

The schematic diagram of the workflow of this study was shown in Figure 1. The work for this study was downloaded from the free public database Gene Expression Omnibus, which included the mRNA expression profile microarray GSE95368 preserved by Fitzgibbons TP et al.^[18]^ This database contained peripheral blood from patients with AMI and controls. Data from this study were processed on the GPL23119 platform, including 18 samples, 12 from female AMI patients and 6 from controls.

### 2.2. Differential gene expression screening

Firstly, we perform data quality checks and normalization using log transformation to eliminate any batches. We employed the “limma” package in R software^[14]^ to screen for DEGs between AMI and controls. Simultaneously, we adjusted *P* value < 0.05 was set as the threshold criterion for statistical significance.

### 2.3. GO and pathway enrichment analysis

In this study, studies on large-scale transcriptional data or genomic data can be usually based on GO analysis. The KEGG pathways database contained information on networks between genes or molecules for genetic research. In this study, DEGs were annotated by GO and KEGG databases. In addition, the Database of Online Tools for Annotation, Visualization, and Integrated Discovery^[15]^ was used to detect GO categories and KEGG pathways. In this study, a *P* value < 0.05 was defined as the threshold for significant enrichment for GO and KEGG analysis. Results were processed and visualized in R software.

### 2.4. Gene set enrichment analysis (GSEA)

We applied GSEA^[16]^ to examine whether the enrichment of the KEGG pathway was statistically significant in female AMI patients and controls. Transcriptomic data was imported into the GSEA desktop application in strict accordance with website instructions. Both *P* < .05 and false discovery rate (FDR) < 0.25 were considered criteria for significant gene sets.

### 2.5. Protein-protein interaction (PPI) network and identification of hub genes

To gain an in-depth understanding of the interactions between proteins encoded by DEGs, we constructed a PPI network of DEGs using the Interaction Gene Retrieval Database.^[17]^ Besides, to accelerate visualizing the statistics generated by the biological network and the integrated Search Tool for the Retrieval of Interacting Genes/Proteins database, we visualized the PPI network using Cytoscape^[18]^ (version 3.7.2) (https://cytoscape.org/). In this study, a composite score >0.5 was considered statistically significant, and the cellular CytoHubba app was used to extract hub genes in this PPI network.

### 2.6. Sample collection

This study was approved by the Medical Ethics Committee of Ordos Central Hospital (approval number: 2022-04-10), following the Declaration of Helsinki. All subjects in this study signed written informed consent. A total of 10 female patients with AMI and 10 controls were included in this study. The diagnosis of AMI was under the Fourth Common Definition of Myocardial Infarction (2018). AMI might be diagnosed as follows: first, when there is clinical evidence of AMI and a rise or fall in cardiac troponin T values with at least 1 value exceeding the 99th percentile reference upper limit, followed by at least the following: patients with symptoms of myocardial ischemia, changes on the electrocardiogram (ECG) indicate new ischemia, there were pathological Q waves on ECG, there was additional evidence for new cardiovascular losses, and angiography or autopsy showed coronary thrombosis. Subjects with no symptoms of myocardial ischemia, no ischemic changes on ECG, and no stenosis on coronary angiography served as controls. Peripheral blood was collected during coronary angiography. Simultaneously, we included subjects with no symptoms of myocardial ischemia, no ischemic changes on ECG, and no stenosis on coronary angiography as controls. Peripheral blood was collected during coronary angiography.

### 2.7. RNA extraction and quantitative RT-PCR

In the study, total RNA from peripheral blood was extracted using a UNlQ-10 column TrizolTotal RNA isolation kit (Sangon Biotech, Shanghai, China) according to the manufacturer’s instructions.

We checked the concentration and purity of the extracted RNA using a NanoDrop 2000 spectrophotometer (Thermo Fisher Scientific, Waltham, MA). Among them, A260/A280 was controlled between 1.8 and 2.0. This study applied Hifair III1st Strand cDNA synthesis SuperMix (Yepsen Biotech, Shanghai, China) for cDNA synthesis. In this study, we used GAPDH as a reference. We performed quantitative RT-PCR by using Hieff qPCR SYBR Green MasterMix (Yepsen Biotech, Shanghai, China) in a Quant Studio 6 Flex system (Thermo Fisher Scientific). Primer sequences (Sangon Biotech) used for reference and candidate genes were shown in Table 1. We applied the 2^−∆∆^Ct method to calculate the relative expression levels of mRNAs.

**Table 1:** Primer sequences.

Gene		Primer sequences
GAPDH	F	GGAGCGAGATCCCTCCAAAAT
	R	GGCTGTTGTCATACTTCTCATGG
S100A9	F	GGTCATAGAACACATCATGGAGG
	R	GGCCTGGCTTATGGTGGTG
MAPK3	F	CTACACGCAGTTGCAGTACAT
	R	CAGCAGGATCTGGATCTCCC
MAPK1	F	TACACCAACCTCTCGTACATCG
	R	CATGTCTGAAGCGCAGTAAGATT
MMP3	F	AGTCTTCCAATCCTACTGTTGCT
	R	TCCCCGTCACCTCCAATCC
IL-17A	F	TCCCACGAAATCCAGGATGC
	R	GGATGTTCAGGTTGACCATCAC
HSP90AB1	F	AGAAATTGCCCAACTCATGTCC
	R	ATCAACTCCCGAAGGAAAATCTC

IL = interleukin, MAPK = mitogen-activated protein kinase.

### 2.8. Statistical analysis

In this study, IBM SPSS 21.0 and GraphPad Prism9 software were used for data analysis. Normally distributed measurement data were expressed as mean ± SD, and abnormal measurement data are the median (25th–75th percentile). Statistical differences between the AMI group and the control group were performed using Student *t* test or Mann–Whitney *U* test in GraphPad Prism9. We completed the construction of the receiver operator characteristic (ROC) curve and the calculation of the area under the ROC curve (AUC) in GraphPad Prism9. Statistical significance was set at *P* < .05.

## 3. Results

### 3.1. Differential gene screening

**Figure 2.**
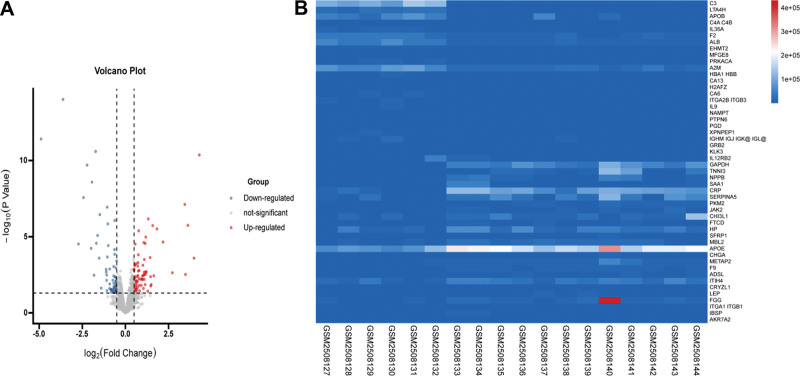
Volcano maps of differentially expressed genes (DEGs). (A)The plot of expression levels of volcanoes. AMI group versus N group. The x-axis represented the fold change in gene expression in the comparison groups as log2, and the y-axis represented −1 × log10 (FDR value) for each DEG. Red dots, blue dots, and gray dots may indicate upregulation, downregulation, and insignificant changes in DEGs. (B) Heatmap of the top 50 DEGs. AMI = acute myocardial infarction, DEGs = differentially expressed genes, FDR = false discovery rate, N = normal person, NS = nonsignificant.

A total of 204 genes were involved in this study, including 120 upregulated genes and 84 downregulated genes. The above genes were compared and defined as DEGs. Relationship between the female AMI group and normal (N) group (Supplementary Table 1, Supplemental Digital Content, http://links.lww.com/MD/I856). Setting parameters were as follows:| log FC| ≥ 0.5, adjust *P* < .05. Volcano plot analysis showed (Fig. 2) that the identification of DEGs could easily distinguish female patients with AMI from normal controls. We selected the top 10 marker genes based on fold change rank and FDR value, respectively. It was found that the top 10 DEGs with the lowest FDR were GAPDH, CRP, APOE, TNNI3, PKM2, JAK2, SAA1, F9, and NPPB and SERPINA5 based on |log FC| ≥ 0.5 between females with AMI and normal subjects. The top 10 DEGs with the highest |log FC| ≤ 0.5 meeting the FDR < 0.05 criterion were C3, LTA4H, ALB, C4A 4B, IL36A, F2, NAMPIT, EHMT2, A2M, MFGE8, and NNAT.

### 3.2. Weighted correlation network analysis

**Figure 3.**
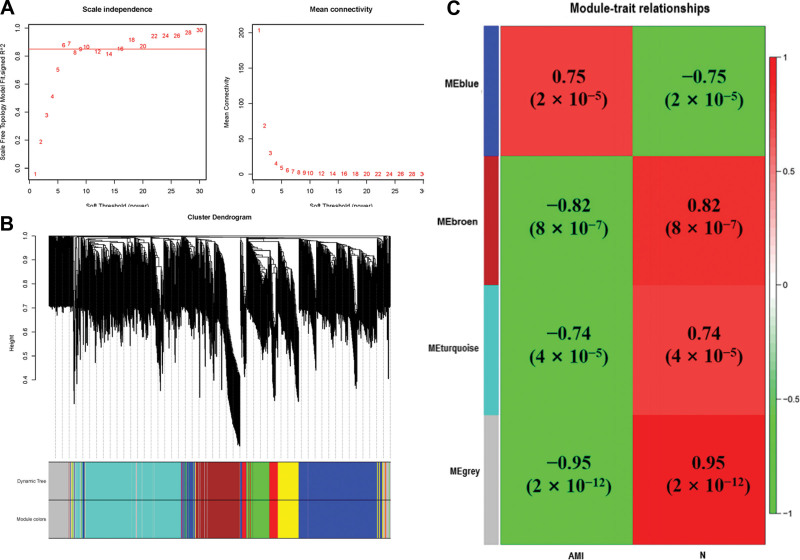
DEGs of WGCNA. (A)Estimation of the soft threshold of a scale-free co-expression network. (B) Cluster tree view of all DEGs. (C) Correlation between each module and AMI patients or control group. AMI = acute myocardial infarction, DEGs = differentially expressed genes, WGCNA = weighted gene co-expression network analysis.

Subsequently, our weighted correlation network analysis package in R software further processed the 204 DEGs and used a soft threshold power of 24 to build a scale-free co-expression network (scale-free *R*^2^ > 0.8). In the subsequent analysis, the soft threshold power *β* was set to 24, as scale independence reached 0.823 (Fig. 3A). DEGs were divided into 4 modules, blue, brown, turquoise, and gray, with the smallest module size ≥30. The clustering dendrogram of DEGs was shown in Figure 3B. We calculated and plotted the correlation between each module and AMI (Fig. 3C). The results revealed that brown (−0.82, *P* < .0001) and blue (0.75, *P* < .0001) were the most negative and positive modules associated with AMI, respectively. Here, the brown modules, including 92 DEGs, were considered key modules related to AMI.

### 3.3. Functional enrichment analysis

**Figure 4.**
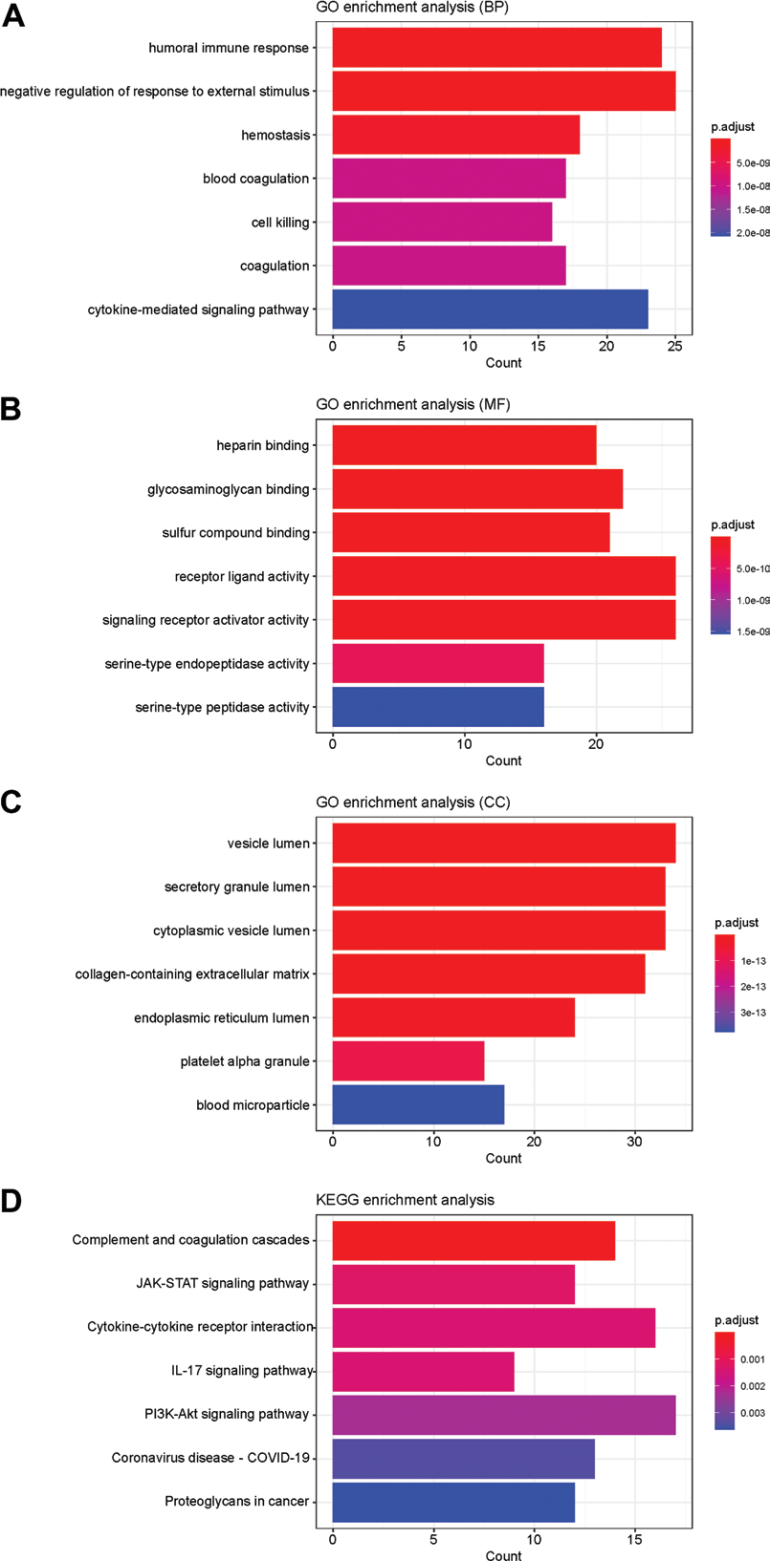
Gene Ontology and KEGG enrichment analysis. (A)Biological process. (B) Cell assembly. (C) Molecular function. (D) KEGG enrichment analysis. KEGG = Kyoto Encyclopedia of Genes and Genomes.

In this study, we performed GO and pathway enrichment analysis on the 204 differentially expressed DEGs using the clusterProfiler tool ^[19]^. GO analysis revealed that DEGs were enriched in 692 biological processes (BP), 57 cellular components (CC), and 30 molecular functions (MF). The first 7 BPs, CCs, and MFs were indicated in Figure 4A–C. Among them, “human immune response,” “heparin-binding” and “vesicle lumen” were significantly enriched in brown modules. KEGG analysis (Fig. 4D) indicated that supplementation and coagulation cascade, just another kinase-signal transducer and activator of transcription, cytokine-cytokine receptor interaction, IL-17, and PI3K-AKT were the main signaling pathways involved in females with AMI.

### 3.4. GSEA analysis

**Figure 5.**
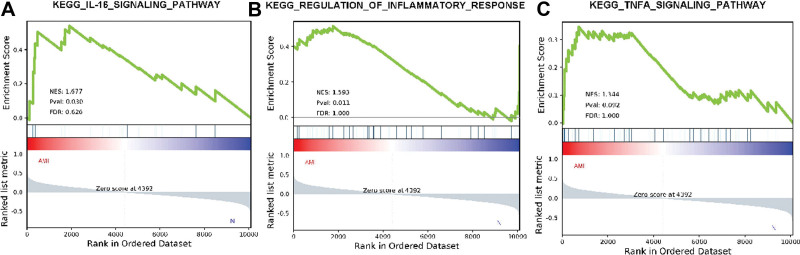
Gene set enrichment analysis. (A)Enrichment map of “IL-16 signaling pathway,” with an enrichment score of 1.677, a *P* value of 0.030, and an FDR value of 0.626. (B) “Regulation of inflammatory response” enrichment map, with an enrichment score of 1.593, a *P* value of 0.011, FDR value of 1.000. (C) Enrichment map of TNFA signaling pathway, with an enrichment score of 1.344, *P* value of 0.092, FDR value of 1.000. FDR = false discovery rate, TNFA = tumor necrosis factor α.

In the study, the distribution of pathway gene sets on all gene expression data from AMI patients and controls was explored using GSEA software (https://www.gsea-msigdb.org/gsea/index.jsp). The results showed that 26/50 genes were upregulated in AMI patients, while 13 gene sets were significantly enriched with FDR < 25%. In the control group, 21/50 gene sets were upregulated and 8 gene sets were highly enriched with FDR < 25%. The IL-16 signaling pathway was significantly enriched in the AMI group with an enrichment score of 1.651 (Fig. 5A). These results suggested that “through the IL-16 signaling pathway” may play a key role in the pathophysiology of AMI in females. The top 3 gene sets shown in Figure 5 are the IL-16 signaling pathway, regulation of inflammatory response (Fig. 5B), and tumor necrosis factor α signaling pathway (Fig. 5C).

### 3.5. PPI analysis of DEGs

**Figure 6.**
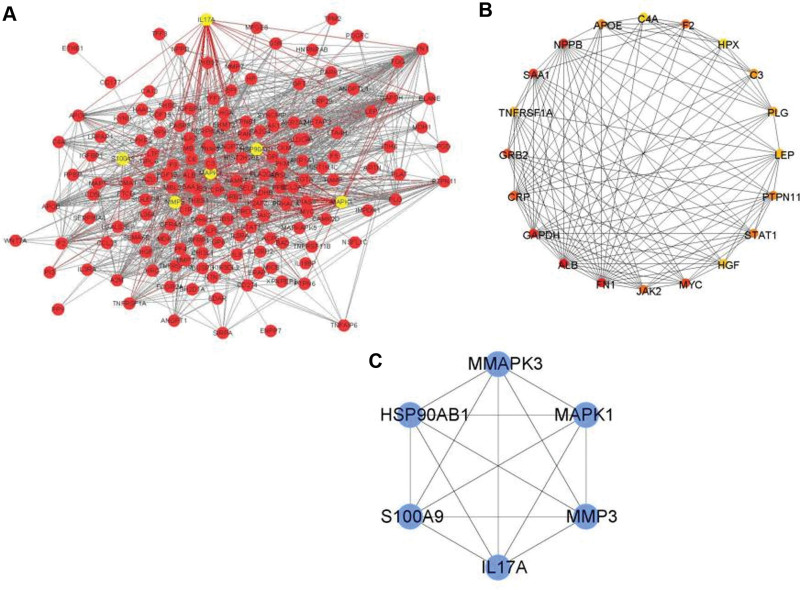
PPI network and hub gene. (A) PPI network. B and C were the 2 most vital module groups. PPI = protein-protein interaction.

To further explore the interactions between AMI and the 204 DEGs compared with N groups, we constructed a PPI network with 54 nodes and 161 edges (Fig. 6A). Using the CytoHubba plug-in, we screened genes with a connectivity degree of >5 in the PPI network analysis as hub genes significantly associated with AMI. As a result, we obtained 2 gene groups, 1 containing the 20 most significant genes, APOE, C4AA, F2, HPX, C3, PLG, LEP, PTPN11, STATA, HGF, MYG, JAK2, FN1, ALB, GAPDH, CRP, GRB2, TNFRSF1A, SAA1, and NPPB (Fig. 6B). Another gene group includes S100A9, mitogen-activated protein kinase (MAPK) 3, MAPK1, MMP3, IL17A, and HSP90AB1 (Fig. 6C).

### 3.6. Baseline characteristics of study subjects

Twenty participants were recruited for this study and divided into 2 groups of females with AMI (n = 10) and a control group (n = 10). The mean age of the AMI group was 60.584 years, while the mean age of the control group was 60.871 years. Both groups were matched in age and gender. The clinical characteristics and laboratory data of all participants are shown in Table 2.

**Table 2:** Clinical characteristics and laboratory data of all participants.

		Females with AMI (n = 10)	Control group (n = 10)	*P* value
	Age	60.584 ± 3.010	60.871 ± 4.099	.556
Vital signs on admission	SBP (mm Hg)	121.50 (114.75–140.50)	120.00 (110.00–140.00)	.868
	DBP (mm Hg)	75.000 (69.500–94.000)	72.000 (70.000–78.000)	.868
	Heart rate (bpm)	78.000 (73.500–87.000)	80.000 (75.000–84.000)	.973
Echocardiographic detection	LVEF (%)	52.500 ± 1.738	63.286 ± 1.848	.001
Blood biochemical indicators	hs-cTnT (ng/mL)	9.930 (8.138–10.000)	0.007 (0.004–0.0100)	.000
	CKMB (U/L)	342.00 (210.25–457.25)	13.00 (9.00–15.00)	.000
	NT-pro-BNP (pg/mL)	682.10 (266.33–894.50)	68.00 (41.70–98.10)	.002
	TC (mmol/L)	5.024 ± 0.199	3.556 ± 0.215	.001
	TG (mmol/L)	1.740 (0.758–2.395)	2.280 (1.880–3.790)	.082
	LDL-C (mmol/L)	3.161 ± 0.155	1.451 ± 0.217	.000
	HDL-C (mmol/L)	1.060 (0.833–1.365)	0.860 (0.780–0.970)	.188
Other comorbidities and smoking history	Hypertension	6 (42.86%)	4 (50%)	.746
	Dyslipidemia	1 (7.14%)	0	NA
	Diabetes	5 (35.71%)	1 (12.5%)	NA
	Smoking or not	8 (57.14%)	2 (25%)	.145

AMI = acute myocardial infarction, CKMB = creatine kinase MB, DBP = diastolic blood pressure, HDL-C = high-density lipoprotein-cholesterol, hs-cTnT = high-sensitivity troponin test, LDL-C = low-density lipoprotein-cholesterol, LVEF = left ventricular ejection fraction, NT-pro-BNP = N-terminal prohormone of brain natriuretic peptide, SBP = systolic blood pressure, TC = total cholesterol, TG = triglycerides.

### 3.7. Validation of hub genes

**Figure 7.**
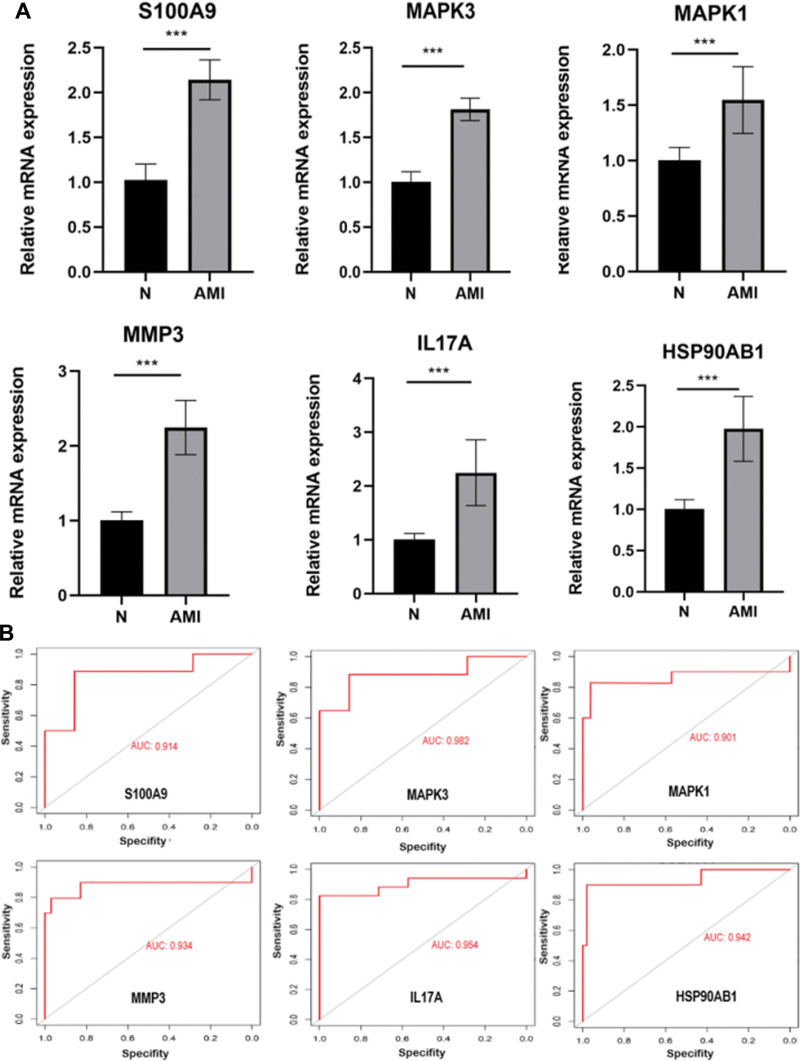
Validation of hub gene. (A–F) Relative expression levels of S100A9, MAPK3, MAPK1, MMP3, IL-17A, and HSP90AB1 (female AMI patients vs normal subjects). (G–L) ROC curves of S100A9, MAPK3, MAPK1, MMP3, IL-17A, and HSP90AB1. AMI = acute myocardial infarction, IL = interleukin, MAPK = mitogen-activated protein kinase, ROC = receiver operator characteristic.

In the analysis based on KEGG and GSEA data, it was found that genes were clustered in terms of inflammatory factors. The enrichment analysis modules S100A9, MAPK3, MAPK1, MMP3, IL17A, and HSP90AB1 were signaling factors in the IL-17 signaling pathway. Therefore, we detected the transcriptional changes of overlapping hub genes S100A9, MAPK3, MAPK1, MMP3, IL17A, and HSP90AB1 in the peripheral blood of female AMI patients and controls by quantitative RT-PCR. The results showed that the expression levels of S100A9, MAPK3, MAPK1, MMP3, IL17A, and HSP90AB1 were all elevated in the AMI group compared with the control group (Fig. 7A and B), which was consistent with the bioinformatics analysis. To evaluate the ability of S100A9, MAPK3, MAPK1, MMP3, IL17A, and HSP90AB1 to discriminate between the AMI group and control group, we applied the ROC curve. The results indicated that the AUCs of S100A9, MAPK3, MAPK1, MMP3, IL17A, and HSP90AB1 were 0.914, 0.982, 0.901, 0.934, 0.954, and 0.942, respectively, revealing that the identified hub genes S100A9, MAPK3, MAPK1, MMP3, IL17A, and HSP90AB1 may have powerful recognition ability, with potential biomarker ability.

## 4. Discussion

In recent years, there has been a worrisome upward trend in hospitalizations for females with MI,^[20]^ and females may be understudied in cardiovascular research. Previous studies have shown that the gene expression of peripheral blood mononuclear cells may be involved in the formation and development of atherosclerosis and MI.^[21]^ Whereas most studies have focused on patients with normal and narrow coronary arteries or patients with AMI and stable coronary heart disease, there might be little evidence that MI can be related to peripheral blood research progress. In this study, we compared gene expression in peripheral blood mononuclear cells from normal coronary arteries and patients with MI based on the high-throughput sequencing gene set GSE95368 in female MI peripheral blood. All 204 DEGs we obtained by the “limma” algorithm were clustered into 4 modules. Then, 92 genes in the brown module were found to be most closely associated with AMI (cor association score = −0.82, *P* < .0001). These genes were mainly enriched in “human immune response,” “heparin-binding” and “vesicle lumen.” GSEA showed that IL-16, regulation of inflammatory response, and tumor necrosis factor α signaling pathways were significant signaling pathways. Finally, the expression of S100A9, MAPK3, MAPK1, MMP3, IL17A, and HSP90AB1 genes in the peripheral blood of female MI patients was verified.

GO terms might include CC, MF, and BP. In the AMI group, only 30 items were significantly enriched in CC, only 57 items were significantly enriched in MF, and up to 692 items were significantly enriched in BP, suggesting that CC, MF, and BP may play a role in the progression and progression of injured myocardium. an important role in repair. By performing GSEA analysis on the gene profiles of GSEs, we obtained a large number of gene sets that were highly enriched in the AMI group. Subsequently, further analysis using the KEGG database and PPI network confirmed that complement and coagulation cascades, just another kinase-signal transducer and activator of transcription signaling pathway, cytokine-cytokine receptor interaction, IL-17 signaling pathway, and PI3K-Akt signaling pathway act on peripheral blood mononuclear cells through, thereby playing a major role in the progression of women with AMI. Furthermore, our enrichment analysis of the GO and KEGG pathways associated with DEGs provided some known and novel molecular mechanisms associated with the development and progression of AMI. The IL-17 signaling pathway was involved in many BP, such as inflammation, immunity, differentiation, cell growth, tumorigenesis, and apoptosis.^[22,23]^ A cross-link between IL-17 signaling and CVD might be established.^[24]^ The emerging evidence suggested that a strong inflammatory response occurs during AMI. We were pleased to find that some vital inflammatory cytokines were enriched in AMI, which was consistent with previous findings.^[25]^

The hub genes with high connectivity included S100A9, MAPK3, MAPK1, MMP3, IL17A, and HSP90AB1. The proteins encoded by the above genes belong to S100A, MAPK, IL superfamily, and heat shock protein 90α family, respectively. The S100 family proteins may have a variety of intracellular and extracellular functions. Previous studies have shown that they interact with a variety of receptors and signal transducers, regulating pathways that control inflammation, cell differentiation, proliferation, energy metabolism, apoptosis, calcium homeostasis, cytoskeleton, and microbial resistance.^[26,27]^ S100 protein has also emerged as a new diagnostic marker for the identification and monitoring of various diseases.^[33]^ The binding of S100 protein to RAGE can activate downstream signaling. Such as transcription factor kappa-B translocation and reactive oxygen species production, which acted as a positive feedback loop for inducing pro-inflammatory phenotypes in multiple cell types including endothelial cells, vascular smooth muscle cells, and leukocytes.^[28]^ The MAPK signaling pathway can be one of the core components of cellular stress, inflammation, differentiation, and apoptosis. Furthermore, the activation of MAPK can be regarded as the final step in the intracellular phosphorylation cascade. Previous studies have reported that MAPK14 is involved in the pathogenesis of numerous inflammatory-driven diseases including atherosclerosis.^[29]^ Some academic research has revealed that IL-1A can encode the IL-1α protein. Knockout of IL-1A in bone marrow cells and application of IL-1α-specific antibodies can inhibit the formation of atherosclerotic plaques in mice.^[30]^ Besides, IL-18 can exhibit similar characteristics in model studies, and the chaperone heat shock protein 90 (HSP90) can be associated with nitric oxide synthase and be crucial for its proper folding and function. The current study might show potential therapeutic value by modulating the relevance of HSP90-nitrous oxide system in various vascular diseases. Some studies have shown that the increased expression of HSP90 leads to increased phosphorylation of GSK3β and decreased phosphorylation of eIF2Bε, thereby aggravating cardiac hypertrophy and the accumulation of HSP90 accelerates vascular remodeling in hypertensive arterial smooth muscle cells.^[31]^

Recently, Boccardi et al^[32]^ showed that there was an interaction between the inflammatory molecule IL-17 and microRNA in male patients with Alzheimer disease, and revealed that miR-122 might be a good candidate regulator. IL-17 mainly played a coordinated role, but it was not concluded that IL-17 was the relevant signal pathway, which was unique to men. In conclusion, whether the role of the IL-17 signaling pathway in AMI is unique to men has not been confirmed by relevant research, which is also the direction of our next efforts.

Compared with previous studies, our work can provide a unique insight into the underlying pathogenesis of AMI. Liu^[33]^ et al obtained 3 microarray datasets (GES, GSE, and GSE) and screened 3 genes FGFBP2, GFOD1, and MLC1 as potential markers for AMI diagnosis. Liu et al^[34]^ performed weighted gene co-expression network analysis assays on GSE4648 to identify genes associated with AMI, ferroptosis, and hypoxia. They used bioinformatics and experimental methods to elucidate the possibility of 10 hub genes (Atf3, Ptgs2, Cxcl1, Socs3, Hspa1b, Selp, Cxcl2, Il1b, Myd88, and S100a8) as diagnostic markers for AMI. In contrast, Zhang et al^[35]^ analyzed the hub genes associated with STEMI and NSTEMI, respectively. They found that STEMI hub genes Aqp1, Armcx1, Gsta4, Hist3h2a, and Il-17 were mainly enriched in cell membrane signal transduction, while Olr1, Nap1l3, Gfer, Dohh, Crispld1, and Ccdc8b as NSTEMI hub genes were significantly associated with energy metabolism. Interestingly, our study mainly focused on the enrichment of inflammatory factor-related gene groups, which provided a new direction for the therapy and prevention of AMI in females.

According to the ROC curve, the AUCs of S100A9, MAPK3, MAPK1, MMP3, IL17A, and HSP90AB1 were 0.914, 0.982, 0.901, 0.934, 0.954, and 0.942, respectively, which indicated that the identified hub genes, including S100A9, MAPK3, MAPK1, MMP3, IL17A, and HSP90AB1 might have strong identification and potential biomarker ability. In general, an AUC of 0.5 indicates no discrimination, 0.7 to 0.8 is acceptable, 0.8 to 0.9 is excellent, and >0.9 is excellent. The AUCs of S100A9, MAPK3, MAPK1, MMP3, IL17A, and HSP90AB1 can all be considered prominent, suggesting that S100A9, MAPK3, MAPK1, MMP3, IL17A, HSP90AB1 may have a strong ability to discriminate female AMI from controls. However, future sample sizes need to be expanded to validate the validity of S100A9, MAPK3, MAPK1, MMP3, IL17A, and HSP90AB1 as biomarkers for AMI in women.

## 5. Limitations

In recent years, dozens of reports have explored the diagnostic value of DEGs for AMI. However, due to the diverse platforms and small sample numbers, the final results might be inconsistent. Similarly, there can be similar shortcomings in this limited observational case-control study. Furthermore, although some DEGs have been identified, the exact relationship between DEGs and the pathophysiological progression of AMI has remained uncertain. The characteristic genes obtained in this paper lack in-depth research. Next, we should carry out further studies of these identified genes. Based on the detected DEGs, we might validate the role of DEGs in the onset and progression of female AMI by exploring their transcription and protein expression and regulation.

## 6. Conclusion

In summary, we reanalyzed the expression profile of GSE95368 with weighted gene co-expression network analysis. The 6 hub genes of S100A9, MAPK3, MAPK1, MMP3, IL17A, and HSP90AB1 in AMI were detected and identified by quantitative RT-PCR. Our report might suggest that the IL-17 signaling pathway and inflammation-related signaling pathways may be potential diagnostic or prognostic genetic markers and therapeutic targets for AMI in females.

## Author contributions

**Conceptualization:** Kun Jiao, Yubao Feng, Changqing Li.

**Data curation:** Kun Jiao, Yubao Feng, Changqing Li.

**Formal analysis:** Yubao Feng, Changqing Li.

**Funding acquisition:** Ping Su.

**Investigation:** Ping Su.

## Supplementary Material


