# Supplementary material for: Bioinformatics analysis and identification of hub genes associated with female acute myocardial infarction patients by using weighted gene co-expression networks
Source: Medicine (Baltimore). 2023 Apr 28;102(17):e33634. doi: 10.1097/MD.0000000000033634 (PMC10145720; doi:10.1097/MD.0000000000033634)
Supplement: Supplementary file 1 [file medi-102-e33634-s001.pdf]

Supplemental Table （Table1） Primer sequences

Gene		Primer sequences
GAPDH	F	GGAGCGAGATCCCTCCAAAAT
	R	GGCTGTTGTCATACTTCTCATGG
S100A9	F	GGTCATAGAACACATCATGGAGG
	R	GGCCTGGCTTATGGTGGTG
MAPK3	F	CTACACGCAGTTGCAGTACAT
	R	CAGCAGGATCTGGATCTCCC
MAPK1	F	TACACCAACCTCTCGTACATCG
	R	CATGTCTGAAGCGCAGTAAGATT
MMP3	F	AGTCTTCCAATCCTACTGTTGCT
	R	TCCCCGTCACCTCCAATCC
IL17A	F	TCCCACGAAATCCAGGATGC
	R	GGATGTTCAGGTTGACCATCAC
HSP90AB1	F	AGAAATTGCCCAACTCATGTCC
	R	ATCAACTCCCGAAGGAAAATCTC
